# Metabolic Chaos After Aneurysmal Subarachnoid Haemorrhage: Longitudinal Glucose–Potassium Ratio Dynamics and Clinical Outcomes

**DOI:** 10.3390/biomedicines14061402

**Published:** 2026-06-22

**Authors:** Adrianna Lebiedzińska, Jarosław Kędziora, Jowita Woźniak, Waldemar Goździk, Małgorzata Burzyńska

**Affiliations:** 1 Clinical Department of Anesthesiology and Intensive Therapy, University Clinical Hospital in Wroclaw, 50-556 Wroclaw, Poland; 2 Clinical Department of Anesthesiology and Intensive Therapy, Faculty of Medicine, Wroclaw Medical University, 50-367 Wroclaw, Poland; jaroslaw.kedziora@umw.edu.pl (J.K.); waldemar.gozdzik@umw.edu.pl (W.G.); malgorzata.burzynska@umw.edu.pl (M.B.); 3 Clinical Department of Neurosurgery, University Centre for Neurology and Neurosurgery, University Clinical Hospital in Wroclaw, 50-556 Wroclaw, Poland; jowita.wozniak@usk.wroc.pl

**Keywords:** subarachnoid hemorrhage, hyperglycemia, glycemic variability, glucose–potassium ratio, neurocritical care, mortality, Vasograde

## Abstract

**Background:** Hyperglycemia after aneurysmal subarachnoid hemorrhage (aSAH) is associated with poor outcome, but admission glucose may not reflect dynamic metabolic stress during neurocritical care. Unlike previous studies focused primarily on admission measurements, we evaluated longitudinal glycemic trajectories and repeated glucose–potassium ratio (GPR) assessment across multiple observation windows in relation to clinical outcomes after aSAH. **Methods:** This retrospective single-center cohort study included 199 consecutive adults with aSAH treated between 2014 and 2025. Serial glucose and potassium measurements obtained during intensive care unit (ICU) stay were used to calculate admission values, longitudinal means across predefined observation windows, glycemic variability, hyperglycemia burden, and GPR. Primary outcomes were 30-day mortality and poor functional outcome at discharge (modified Rankin Scale ≥ 3). Secondary outcomes included delayed cerebral ischemia (DCI), delayed neurological deterioration (DND), transcranial Doppler (TCD) vasospasm, neurological deficit at ICU discharge, and length of stay. **Results:** Thirty-day mortality occurred in 35 patients (17.6%). Longitudinal metabolic markers demonstrated stronger associations with outcomes than admission values. Mean 30-day GPR was independently associated with mortality (OR 2.56, 95% CI 1.66–4.16; *p* < 0.001) and poor functional outcome (OR 2.90, 95% CI 1.80–5.03; *p* < 0.001). Hyperglycemia burden was associated with mortality (OR 1.10 per additional hyperglycemic day, 95% CI 1.02–1.20; *p* = 0.020) and poor functional outcome (OR 1.39, 95% CI 1.19–1.71; *p* < 0.001). Early GPR during the early brain injury period was associated with DCI (OR 1.40, 95% CI 1.01–1.93; *p* = 0.043), whereas 30-day GPR was associated with DND (OR 1.47, 95% CI 1.08–2.07; *p* = 0.019). ICU-specific GPR was associated with neurological deficit at ICU discharge (OR 2.06, 95% CI 1.29–3.50; *p* = 0.004), but not with TCD-defined vasospasm. Addition of GPR improved mortality prediction compared with the clinical model alone (AUC 0.86 vs. 0.77; *p* = 0.002). **Conclusions:** Longitudinal metabolic dysregulation after aSAH is strongly associated with mortality and neurological outcomes. Persistent hyperglycemia and repeated GPR assessment provide prognostic information beyond admission glucose, with early abnormalities associated with DCI and sustained disturbances linked to mortality and disability.

## 1. Introduction

Aneurysmal subarachnoid hemorrhage (aSAH) is a severe form of hemorrhagic stroke associated with high mortality and long-term disability despite advances in neurocritical care and aneurysm treatment [1,2,3]. Although relatively uncommon, aSAH disproportionately affects younger adults and carries substantial social and economic burden [1,4]. Clinical outcome depends not only on the initial hemorrhage severity, but also on secondary neurological and systemic complications developing during intensive care treatment [5,6].

Traditionally, delayed cerebral ischemia (DCI) and cerebral vasospasm were considered the principal causes of delayed neurological deterioration (DND) after aSAH [6,7]. However, contemporary research increasingly emphasizes the role of early brain injury (EBI), characterized by transient global ischemia, blood–brain barrier disruption, inflammation, oxidative stress, and microcirculatory dysfunction occurring within the first 72 h after aneurysm rupture [8,9,10]. In parallel, aSAH induces a systemic neuroendocrine stress response involving sympathetic and hypothalamic–pituitary–adrenal axis activation, contributing to disturbances in glucose metabolism, electrolyte balance, and cardiovascular homeostasis [11,12].

Hyperglycemia is among the most common metabolic abnormalities after aSAH and has consistently been associated with worse neurological severity, mortality, DCI, and poor functional outcome [13,14,15,16,17]. However, admission glucose represents only a single time-point during a dynamic critical illness. In critically ill populations, longitudinal glucose exposure and glycemic variability may provide greater prognostic value than isolated baseline measurements [18,19], and similar observations have recently been reported in aSAH [20,21,22,23].

The glucose–potassium ratio (GPR) has recently emerged as a potential composite biomarker reflecting both stress hyperglycemia and neuroendocrine-mediated potassium shifts [24,25]. Previous studies demonstrated associations between elevated admission GPR and poor outcome after aSAH [26]. However, evidence regarding repeated longitudinal GPR assessment during prolonged neurocritical care remains limited [23,26].

We therefore evaluated whether dynamic glycemic indices and repeated GPR measurements are associated with clinically relevant outcomes after aSAH. We hypothesized that longitudinal metabolic markers would outperform admission measurements for prediction of mortality and neurological outcome.

## 2. Materials and Methods

### 2.1. Study Design

Adult patients with aSAH treated in the Intensive Care Unit (ICU) of the University Clinical Hospital in Wroclaw, Poland, between January 2014 and January 2025 were retrospectively enrolled in this single-center cohort study. The diagnosis of aSAH was established on the basis of clinical presentation and confirmed by neuroimaging using non-contrast head computed tomography (CT), CT angiography (CTA), or digital subtraction angiography (DSA) [2,3].

Eligible patients were required to be aged ≥18 years, admitted within 24 h of symptom onset, and treated with definitive aneurysm occlusion (microsurgical clipping or endovascular coiling) within 48 h after admission. Exclusion criteria were: non-aneurysmal SAH, pre-existing severe central nervous system disease unrelated to aSAH, pregnancy, major procedural complications, clinical evidence of nonsurvivable brain injury at presentation, and incomplete clinical or laboratory data required for analysis.

**Figure 1:**
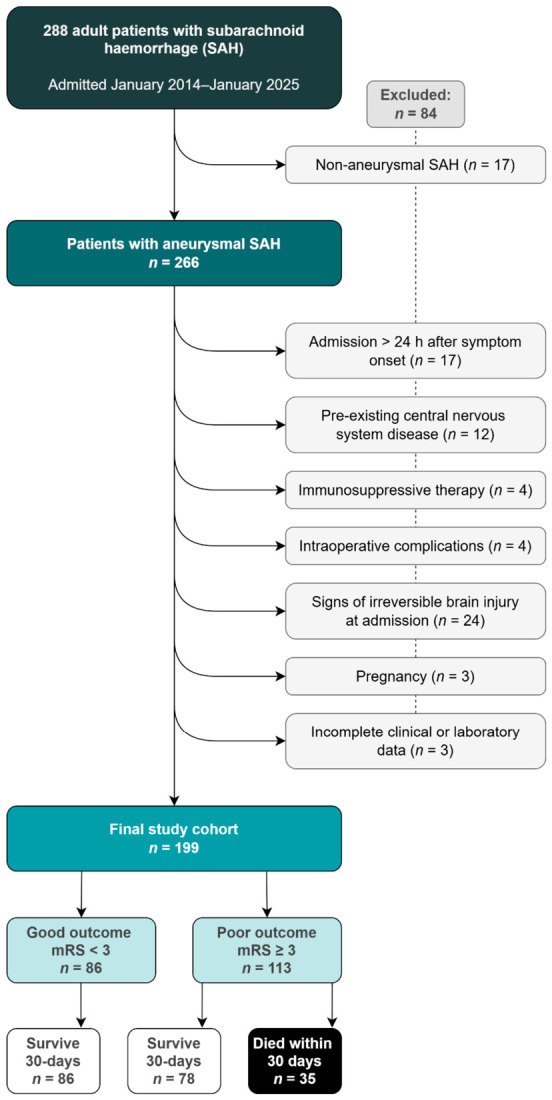
Flowchart of patient selection and study cohort formation.

The study population selection process is presented in Figure 1.

### 2.2. Clinical Management

Patient management followed institutional neurocritical care practices consistent with contemporary American Heart Association/American Stroke Association recommendations for aSAH. Core management principles included early aneurysm securing, routine nimodipine administration, hemodynamic and respiratory optimization, prevention of secondary brain injury, and surveillance for DCI [3,27].

Hyperglycemia was defined as glucose >10 mmol/L (180 mg/dL), and hypoglycemia as glucose <4 mmol/L (70 mg/dL) [15,16], and was managed according to contemporary critical care recommendations targeting avoidance of severe hyperglycemia while minimizing hypoglycemia. Intravenous or subcutaneous insulin therapy was used at the discretion of the treating team according to ICU protocols [28,29].

In patients with pre-existing diabetes mellitus, glycemic management was individualized, including continuation or adjustment of chronic antihyperglycemic therapy in accordance with the annual Standards of Care in Diabetes issued by the American Diabetes Association Professional Practice Committee.

Transcranial Doppler ultrasonography (TCD) was performed daily or every second day as part of routine vasospasm surveillance.

TCD-defined vasospasm was diagnosed using established velocity criteria based on middle cerebral artery mean flow velocity and the Lindegaard ratio [30]. When elevated flow velocities suggested vasospasm, confirmatory vascular imaging using CTA or DSA was obtained. Subsequent management could include hemodynamic augmentation, induced hypertension when appropriate, balloon angioplasty, and/or intra-arterial vasodilator therapy.

DCI was defined according to the multidisciplinary consensus criteria proposed by Vergouwen et al. as a new focal neurological deficit, decrease in consciousness, or new cerebral infarction not attributable to other causes after aneurysm occlusion [7].

DND was defined as delayed worsening in neurological status during hospitalization not fully explained by sedation or immediate postoperative state, including deterioration related to vasospasm, hydrocephalus, seizures, infection, metabolic disturbances, or evolving ischemia [6,7]. Because this endpoint reflects global in-hospital neurological worsening, final classification was based on the last structured neurological assessment before hospital discharge.

Neurological deficit at ICU discharge was defined as persistence of clinically relevant focal or global neurological impairment at the time of ICU discharge.

### 2.3. Data Collection

Clinical, laboratory, treatment, and outcome data were retrospectively extracted from electronic medical records and the institutional neurocritical care database.

Baseline variables included age, sex, body mass index (BMI), smoking status, arterial hypertension, diabetes mellitus, and treatment modality. Neurological severity at admission was assessed using the Glasgow Coma Scale (GCS), Hunt–Hess grade, World Federation of Neurosurgical Societies (WFNS) scale, modified Fisher grade, and Vasograde [31,32,33].

Vasograde was derived according to the previously published classification and stratified patients into three categories: Vasograde-Green (modified Fisher grade 1–2 and WFNS grade 1–2), Vasograde-Yellow (modified Fisher grade 3–4 and WFNS grade 1–3), and Vasograde-Red (WFNS grade 4–5 irrespective of modified Fisher grade) [32,33].

Aneurysm location was categorized as anterior or posterior circulation. Admission laboratory variables included plasma glucose, serum potassium, white blood cell (WBC) count, hemoglobin, hematocrit, and C-reactive protein (CRP).

During ICU stay, glucose and potassium measurements were obtained at least once daily according to the institutional monitoring protocol using either the central laboratory analyzer or point-of-care blood gas analyzers.

### 2.4. Outcomes

The primary outcomes were 30-day in-hospital mortality and poor functional outcome at hospital discharge, defined as modified Rankin Scale (mRS) ≥3. Secondary outcomes included DCI, DND, TCD-defined vasospasm, neurological deficit at ICU discharge, and ICU and hospital length of stay (LOS).

### 2.5. Glycemic Variables

Admission glucose and potassium were defined as the first available measurements obtained after hospital admission. GPR was calculated as glucose divided by potassium concentration (both expressed in mmol/L). In patients with available glycated hemoglobin (HbA1c) data, the stress hyperglycemia ratio (SHR) was calculated using estimated average glucose derived from HbA1c values.

Longitudinal metabolic exposure was assessed using mean glucose and mean GPR calculated across predefined observation windows: admission, EBI (0–3 days), 7 days, 14 days, 21 days, and 30 days. The 30-day window was considered the principal measure of cumulative metabolic exposure. ICU-specific GPR was additionally calculated across the duration of ICU stay.

Glycemic variability during hospitalization was assessed using glucose standard deviation (SD), glucose range (maximum–minimum), and mean daily glucose change. GPR variability was defined as the standard deviation of serial GPR values.

Hyperglycemia burden was evaluated using the number of ICU days with hyperglycemia, the percentage of ICU days with hyperglycemia, and the maximum number of consecutive hyperglycemic days.

### 2.6. Statistical Analysis

All statistical computations were performed using R version 4.5.2 (R Foundation for Statistical Computing, Vienna, Austria) and RStudio version 2026.1.2.418 (Posit Software, PBC, Boston, MA, USA).

Continuous variables are presented as median with interquartile range (IQR), and categorical variables as counts with percentages. Group comparisons were performed using the Mann–Whitney U test for continuous variables and the chi-square test or Fisher’s exact test for categorical variables, as appropriate.

Binary outcomes were analyzed using multivariable logistic regression and reported as odds ratios (ORs) with 95% confidence intervals (CIs). Time-to-death analyses were performed using Cox proportional hazards models, whereas ICU and hospital LOS were analyzed using linear regression after logarithmic transformation. Continuous predictors were standardized (z-scores), and effect estimates are reported per 1 standard deviation increase.

For primary outcomes (30-day mortality, poor functional outcome, neurological deficit at ICU discharge, ICU and hospital LOS), models were adjusted for age, GCS, and Hunt–Hess grade. Models for DCI, DND, and TCD-defined vasospasm were adjusted for age and Vasograde category. In Cox proportional hazards analyses, adjustment was limited to age and Hunt–Hess grade to avoid overfitting.

To align metabolic exposure with outcome timing, EBI GPR (0–3 days) was used for analyses of DCI, whereas 30-day GPR was used for DND and functional outcomes. ICU-specific GPR was used for analyses of neurological deficit at ICU discharge.

Prognostic performance was evaluated using receiver operating characteristic (ROC) curves with calculation of the area under the curve (AUC). Differences between ROC curves were assessed using DeLong’s test.

Sensitivity analyses included additional adjustments for diabetes mellitus, hypertension, and smoking status, as well as analyses restricted to patients without diabetes mellitus. Statistical significance was defined as a two-tailed *p*-value below 0.05.

## 3. Results

### 3.1. Patient Characteristics

A total of 199 patients with aSAH met the eligibility criteria and were included in the final analysis. Thirty-day in-hospital mortality occurred in 35 patients (17.6%).

The median age of the cohort was 56 years (IQR 47–66), and 56.8% of patients were female. Female patients were older (58 [48–68] vs. 54 [45–65] years, *p* = 0.037) and had lower BMI values (24.8 [23.0–28.3] vs. 27.8 [24.7–30.4] kg/m^2^, *p* < 0.001). Overall disease severity was substantial, with 43.7% of patients presenting with poor Hunt–Hess grade and 44.2% classified as Vasograde red.

Compared with survivors, non-survivors were older and presented with more severe neurological injury, including worse Hunt–Hess grade, lower GCS, and higher Vasograde category (all *p* ≤ 0.030). Admission glucose and admission GPR were also significantly higher among non-survivors (both *p* < 0.001). Posterior circulation aneurysms were more frequent in non-survivors (28.6% vs. 12.8%, *p* = 0.036). No significant between-group differences were observed for sex, BMI, treatment modality, smoking status, diabetes mellitus, hypertension, admission potassium, WBC count, or CRP.

Multiple aneurysms were observed more frequently among women (38% vs. 19%, *p* = 0.003), and TCD-defined vasospasm also occurred more commonly among women (64% vs. 48%, *p* = 0.030). However, no significant sex-related differences were identified for admission glucose concentration and GPR, longitudinal glycemic indices, longitudinal GPR measures, occurrence of DCI or DND, mortality, or poor functional outcome. Baseline characteristics are summarized in Table 1.

Regarding hospital disposition, 46.7% of patients were discharged home, 23.1% to rehabilitation facilities, 8.5% to long-term care institutions, and 0.5% to another neurology ward, while 21.1% died during hospitalization.

### 3.2. Metabolic Profile During ICU Stay

The cohort demonstrated substantial heterogeneity in metabolic trajectories during neurocritical care. Median admission glucose was 8.5 mmol/L (IQR 7.0–10.4), whereas median admission GPR was 2.3 (IQR 1.9–3.0). Both markers were significantly higher in non-survivors than survivors (Table 1).

Across the 30-day observation window, median mean glucose was 7.4 mmol/L (IQR 6.7–8.3), while median mean GPR was 1.8 (IQR 1.7–2.0). During the EBI period, metabolic stress was numerically greater, with higher glucose and GPR values than later windows.

Hyperglycemia during ICU stay was common. The median number of ICU days with at least one glucose value >10 mmol/L was 1 day (IQR 0–3), whereas hypoglycemia was uncommon, with a median of 0 days (IQR 0–0).

In adjusted models for 30-day mortality, both admission and longitudinal markers were associated with adverse outcomes. However, effect sizes were stronger for persistent measures, particularly mean 30-day GPR, longitudinal mean glucose, glycemic variability, and cumulative hyperglycemia burden (Table 2).

### 3.3. Primary Outcomes

#### 3.3.1. Thirty-Day In-Hospital Mortality and Survival Analysis

Higher mean 30-day GPR was independently associated with increased 30-day in-hospital mortality in multivariable logistic regression models adjusted for age and baseline neurological severity. Each 1 SD increase in mean GPR was associated with a 2.56-fold higher odds of death (OR 2.56, 95% CI 1.66–4.14, *p* < 0.001).

**Figure 2:**
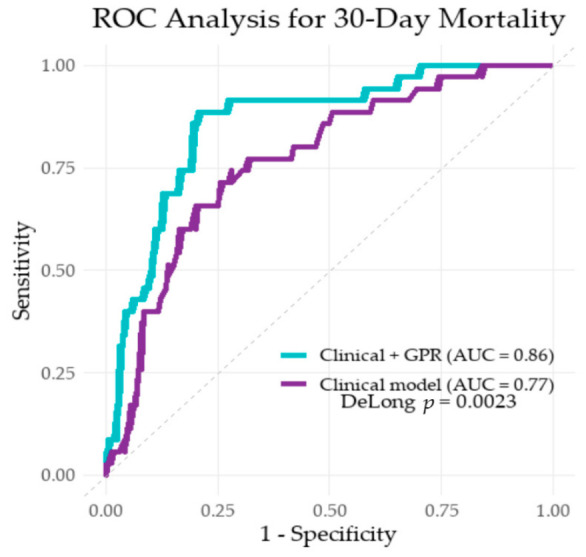
Receiver operating characteristic (ROC) analysis for prediction of 30-day mortality after aneurysmal subarachnoid hemorrhage. The addition of the glucose–potassium ratio (GPR) to the clinical model improved discriminative performance for 30-day mortality (AUC 0.86 vs. 0.77; DeLong *p* = 0.0023). The clinical model included age and Hunt–Hess grade.

Inclusion of mean 30-day GPR significantly improved model discrimination for 30-day mortality. The area under the receiver operating characteristic curve (AUC) increased from 0.77 to 0.86 after adding GPR to the clinical model including age and Hunt–Hess grade. The difference in AUC was statistically significant (DeLong test, *p* = 0.003). ROC curves comparing the clinical model alone and the clinical model supplemented with GPR for prediction of 30-day mortality are presented in Figure 2. 

After adjustment for age and Hunt–Hess grade in the Cox proportional hazards model, higher mean 30-day GPR continued to demonstrate an independent association with mortality risk (HR 1.90 per 1 SD increase, 95% CI 1.49–2.44; *p* < 0.001). Hunt–Hess grade was also independently associated with mortality (HR 1.65, 95% CI 1.20–2.26; *p* = 0.002), whereas age did not reach statistical significance.

HbA1c was available in 51 patients (25.6%). The median SHR was 1.32 (IQR 1.17–1.66). Patients with and without HbA1c measurements did not differ materially in age or mortality. In the subgroup with available HbA1c measurements, SHR demonstrated modest univariable discrimination for 30-day mortality (AUC 0.67, 95% CI 0.48–0.87), but did not remain independently related to mortality after multivariable adjustment (OR 0.96, 95% CI 0.42–2.26; *p* = 0.917). Addition of SHR to the clinical model did not improve predictive discrimination (AUC 0.80 vs. 0.80; DeLong *p* = 0.848).

#### 3.3.2. Poor Functional Outcome at Hospital Discharge

Poor functional outcome (mRS ≥ 3) was observed in 113 patients (56.8%).

Several metabolic markers were independently related to disability at discharge, with persistent hyperglycemia burden and longitudinal GPR demonstrating the strongest relationships. Mean 30-day GPR was independently associated with poor functional outcome at discharge. Each 1 SD increase in GPR was associated with a significantly higher odds of poor outcome (OR 2.90, 95% CI 1.80–5.03, *p* < 0.001).

Glycemic variability was also associated with poor outcome, whereas isolated admission markers showed weaker and less consistent effects than repeated longitudinal measures.

Each additional hyperglycemic ICU day was associated with a 39% increase in the odds of poor functional outcome (OR 1.39, 95% CI 1.19–1.71; *p* < 0.001), whereas each additional consecutive hyperglycemic day was associated with nearly twofold higher odds of poor outcome (OR 1.88, 95% CI 1.41–2.70; *p* < 0.001).

### 3.4. Secondary Outcomes

#### 3.4.1. DCI, DND and TCD Confirmed Vasospasm

DCI occurred in 88 patients (44.2%). Higher mean GPR during the EBI period was independently associated with DCI after adjustment for age and Vasograde (OR 1.40 per 1 SD increase, *p* < 0.05).

DND was observed in 100 patients (50.3%). Early glucose and GPR measures showed borderline relationships, whereas stronger associations emerged for later longitudinal GPR windows.

Higher mean 30-day GPR was independently associated with DND after adjustment for age and Vasograde (OR 1.40 per 1 SD increase).

Vasospasm seen in TCD was common during hospitalization, occurred in 113 patients (56.8%). No significant association was observed between mean GPR and TCD-defined vasospasm (OR 1.11, 95% CI 0.47–2.65; *p* = 0.813).

#### 3.4.2. Neurological Deficit at ICU Discharge

Neurological deficit at ICU discharge was present in 93 of 167 evaluable patients (55.7%).

Admission markers were not strongly predictive, whereas repeated glucose and GPR measurements across EBI, 7-day, 14-day, 21-day, and 30-day windows were consistently associated with persistent neurological deficit. Mean ICU GPR was strongly associated with neurological deficit at ICU discharge. Each 1 SD increase was associated with more than twofold higher odds of deficit (OR 2.15, *p* < 0.001).

Predictive performance increased progressively across longer observation windows, with the strongest associations observed for ICU and 30-day GPR measures.

#### 3.4.3. ICU and Hospital LOS

Burden-based hyperglycemia measures were associated with prolonged ICU stay, including cumulative hyperglycemic days and consecutive hyperglycemic streaks.

In contrast, mean GPR was not independently associated with ICU LOS after adjustment. However, hyperglycemia burden metrics remained significantly associated with prolonged ICU stay.

Each additional hyperglycemic ICU day was associated with an approximately 4.6% longer ICU stay after adjustment for age and neurological severity.

#### 3.4.4. In-Hospital Mortality and Time to Death

Overall in-hospital mortality was 21.1% (42/199), including 7 additional deaths occurring after day 30. Among non-survivors, median time to death was 12.5 days (IQR 9.3–19.8). Most deaths occurred between days 8 and 30 (69.0%).

Associations between GPR measures and secondary clinical outcomes are summarized in Table 3. 

### 3.5. Relationship Between Hemorrhage Severity and Metabolic Indices

A clear severity-dependent metabolic gradient was observed across Vasograde categories.

Admission glucose increased progressively from 7.5 mmol/L in the green group to 9.7 mmol/L in the red group (*p* for trend < 0.001). The same stepwise pattern was observed for mean glucose during the EBI period and across the 30-day observation window (all *p* for trend < 0.001).

Admission GPR increased from 2.13 in Vasograde green to 2.88 in red patients (*p* for trend < 0.001).

**Figure 3:**
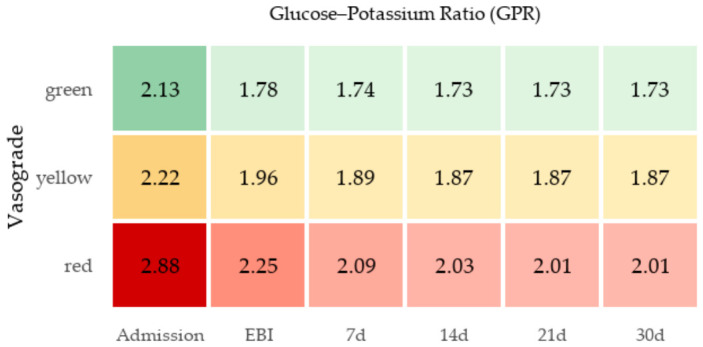
Temporal evolution of the glucose–potassium ratio (GPR) across Vasograde categories. Heatmap demonstrating mean GPR values from admission through the 30-day observation period according to Vasograde classification. Increasing color intensity corresponds to higher GPR values. EBI = early brain injury (days 1–3); d = days.

Similarly, GPR demonstrated a persistent linear relationship with hemorrhage severity. Admission GPR was highest in patients classified as red Vasograde, and elevated values remained evident throughout subsequent observation windows. Mean GPR during EBI, 7-day, 14-day, 21-day, and 30-day windows increased progressively from green to yellow to red categories (all *p* for trend < 0.001). The temporal evolution of the GPR across Vasograde categories is presented in Figure 3.

Hyperglycemia burden demonstrated a strong association with adverse outcomes. Each additional ICU day with hyperglycemia was associated with a 10% increase in the odds of 30-day mortality (OR 1.10, 95% CI 1.02–1.20; *p* = 0.020) and a 39% increase in the odds of poor functional outcome at discharge (OR 1.39, 95% CI 1.19–1.71; *p* < 0.001).

### 3.6. Aneurysm Location and Metabolic Indices

Patients with posterior circulation aneurysms demonstrated consistently higher metabolic stress markers than those with anterior circulation aneurysms.

Median mean GPR during the EBI period was higher in the posterior group and approached statistical significance (2.15 vs. 1.95; *p* = 0.053). Thereafter, the difference became significant and persisted across all subsequent observation windows: 7 days (2.14 vs. 1.84; *p* = 0.005), 14 days (2.04 vs. 1.82; *p* = 0.004), 21 days (2.03 vs. 1.81; *p* = 0.005), and 30 days (2.03 vs. 1.8; *p* = 0.006).

Similarly, glucose concentrations were higher in patients with posterior aneurysms during the EBI period (8.39 vs. 7.72 mmol/L; *p* = 0.048) and across the 30-day window (8.22 vs. 7.28 mmol/L; *p* = 0.003).

### 3.7. Sensitivity Analyses

The primary findings remained robust across predefined sensitivity analyses. Additional adjustment for diabetes mellitus, hypertension, and smoking status did not materially alter the association between longitudinal GPR and 30-day mortality. In the fully adjusted model, mean 30-day GPR remained independently associated with mortality (OR 2.92, 95% CI 1.79–5.13; *p* < 0.001).

In analyses restricted to patients without diabetes mellitus, mean 30-day GPR also remained independently associated with mortality (OR 2.64, 95% CI 1.59–4.70; *p* < 0.001).

Stratified analyses according to aneurysm location and treatment modality demonstrated similar effect directions in anterior and posterior circulation aneurysms as well as in patients treated with endovascular coiling or surgical clipping, although precision was reduced in smaller subgroups. Overall, these analyses supported the robustness of the primary findings.

## 4. Discussion

### 4.1. Principal Findings

Longitudinal metabolic dysregulation was consistently associated with clinically relevant outcomes in our cohort. While admission hyperglycemia retained prognostic value, repeated measurements during ICU stay demonstrated stronger and more consistent associations with 30-day mortality, poor functional outcome, and neurological recovery. These observations further suggest that metabolic response after aSAH is a dynamic, time-dependent process rather than a static abnormality captured at admission.

Female patients demonstrated a higher prevalence of TCD-defined vasospasm, which remained significant after adjustment for multiple aneurysms, suggesting possible sex-related differences in cerebrovascular reactivity following aSAH.

Among the evaluated indices, persistent hyperglycemia burden and longitudinal GPR showed the most robust associations with mortality and disability. Markers of instability, including glycemic variability and day-to-day glucose changes, were also independently associated with adverse outcomes, indicating that both magnitude and fluctuation of metabolic stress are clinically relevant.

Importantly, prognostic performance improved with longer observation windows. In particular, mean 30-day GPR significantly improved discrimination beyond clinical severity models, highlighting the added value of longitudinal metabolic monitoring in a disease characterized by delayed complications and prolonged ICU care.

### 4.2. Interpretation of Findings

#### 4.2.1. Persistent Hyperglycemia as a Marker of Ongoing Injury

Acute aneurysm rupture induces a profound neuroendocrine stress response mediated by sympathetic activation and hypothalamic–pituitary–adrenal axis stimulation, resulting in rapid onset of stress hyperglycemia. Persistent hyperglycemia likely reflects sustained systemic stress, ongoing brain injury, or both.

Beyond being a marker, hyperglycemia may contribute directly to secondary injury through oxidative stress, endothelial dysfunction, inflammation, and impaired cerebrovascular autoregulation. In this context, cumulative exposure—rather than isolated peaks—appears particularly relevant. Our findings that hyperglycemia burden and prolonged hyperglycemic streaks were associated with adverse outcomes support this interpretation.

#### 4.2.2. Glycemic Variability and Metabolic Instability

Glycemic variability emerged as an independent prognostic signal. Fluctuations in glucose may induce greater cellular stress than sustained elevations by amplifying oxidative and inflammatory responses. Unlike stable hyperglycemia, rapid oscillations may impair adaptive cellular mechanisms and exacerbate vascular dysfunction [18,19].

Our results extend previous observations by demonstrating that simple trajectory-based measures, such as daily glucose change and consecutive hyperglycemic days, capture clinically meaningful instability. These findings suggest that future management strategies should consider not only absolute glucose levels but also stability of glycemic control [22,23].

In analyses restricted to patients without diabetes mellitus, repeated GPR and cumulative hyperglycemia measures remained significantly associated with 30-day mortality and poor functional outcome, indicating that the observed relationships were not driven solely by pre-existing dysglycemia.

#### 4.2.3. GPR as a Composite Marker of Neuroendocrine Stress

The GPR integrates disturbances in glucose metabolism and potassium homeostasis, both of which are influenced by neuroendocrine activation after aSAH. Potassium shifts reflect catecholamine excess, insulin effects, and systemic stress, making GPR a biologically composite marker [11,12].

Consistent with prior studies, admission GPR was associated with outcome; however, our data demonstrate that repeated GPR measurements provide superior prognostic value. The progressive increase in predictive performance across time windows suggests that GPR reflects sustained physiological stress rather than a single acute response [26].

Although glucose alone showed comparable discriminative performance, GPR offers additional pathophysiological insight by incorporating electrolyte imbalance, supporting its role as a clinically useful composite biomarker [24].

The observed stepwise increase in glucose and GPR across Vasograde categories further supports the relationship between metabolic dysregulation and initial hemorrhage severity. Importantly, this gradient persisted across subsequent observation windows, suggesting that severe early neurological injury may initiate prolonged systemic metabolic stress extending beyond the admission period.

Glucose and potassium concentrations were managed according to contemporary ICU protocols targeting avoidance of severe hyperglycemia and electrolyte disturbances. Despite standardized metabolic management, longitudinal GPR measures remained strongly associated with mortality and neurological outcomes. This observation may suggest that GPR reflects the overall severity of neuroendocrine and systemic stress responses after aSAH rather than merely uncontrolled metabolic imbalance.

#### 4.2.4. Temporal Dissociation of Early and Late Outcomes

A key observation was the temporal divergence between predictors of DCI and predictors of mortality and disability. Early metabolic disturbances were associated with DCI, whereas longitudinal abnormalities were more strongly related to mortality and functional outcomes.

This pattern is biologically plausible. Early metabolic dysregulation may reflect vulnerability to delayed ischemic injury through mechanisms such as inflammation, microvascular dysfunction, and impaired autoregulation [6,8,10]. In contrast, mortality and disability are influenced by cumulative exposure to systemic stress, complications, and prolonged critical illness, which are better captured by longitudinal markers.

Interestingly, longitudinal GPR was associated with DCI and DND but not with TCD-defined vasospasm. This finding supports the contemporary view that delayed neurological injury after aSAH is not solely driven by large-vessel vasospasm, but also involves microcirculatory dysfunction, inflammation, and systemic metabolic stress.

The lack of a significant independent relationship between longitudinal GPR dynamics and ICU length of stay may reflect the influence of multiple overlapping clinical pathways affecting patient outcomes. Patients with the most pronounced metabolic dysregulation often died early during hospitalization, potentially resulting in shorter ICU stays despite greater disease severity. Accordingly, ICU length of stay may incompletely capture the burden of critical illness in rapidly deteriorating patients.

### 4.3. Comparison with Previous Studies

Our findings align with prior studies demonstrating associations between admission hyperglycemia and poor outcomes after aSAH [13,15,16,17,34]. However, consistent with emerging evidence, longitudinal measures and glycemic variability appear to provide greater prognostic value than single baseline measurements [21,22,23,32].

Previous work has also highlighted the importance of glucose variability and cumulative exposure, as well as the potential prognostic role of GPR [22,26]. Our study extends these observations by integrating multiple complementary metrics—including burden, variability, and trajectory-based indices—and by demonstrating consistent associations across a range of clinically relevant outcomes.

A separate aspect concerns the SHR, which has recently been proposed as a marker distinguishing acute stress-induced hyperglycemia from chronic dysglycemia by relating admission glucose to estimated baseline glycemic status derived from HbA1c. Previous studies suggested that stress-related hyperglycemia may be more strongly associated with neurological severity and adverse outcomes after aSAH than chronic glycemic abnormalities alone [35,36]. In our cohort, SHR showed only moderate ability to identify patients at risk of 30-day mortality, and its contribution became negligible when considered alongside conventional risk factors. Addition of SHR to the clinical model also did not improve discriminatory performance. These findings may reflect the limited HbA1c subgroup available for analysis, but they also suggest that longitudinal glycemic burden and repeated metabolic measurements may capture the systemic response to early brain injury more comprehensively than a single admission-based stress index.

### 4.4. Relevance to Patient Management

These results may carry important implications for neurocritical care practice.

First, they support a shift from single-point measurements toward longitudinal metabolic monitoring. Because risk evolves over time after aSAH, repeated assessment may better identify patients with persistent physiological instability.

Second, the identified markers are readily available from routine laboratory data and could be integrated into clinical workflows, including automated monitoring systems or ICU dashboards.

Third, metabolic markers should complement—not replace—established neurological severity scales. While clinical scales reflect initial hemorrhage severity [31], longitudinal metabolic measures may capture ongoing systemic stress and treatment response.

Finally, whether targeting glycemic variability or cumulative hyperglycemia improves outcomes remains uncertain and warrants prospective investigation [14].

### 4.5. Limitations

The findings of this study should be interpreted within the context of several methodological constraints. First, the retrospective single-center design limits causal inference and may reduce the generalizability of the findings. In addition, glucose and potassium measurements were not obtained according to a fully standardized sampling schedule and may have been influenced by the patients’ clinical condition and treating physicians’ decisions. Consequently, ascertainment bias and unequal sampling intensity between patients cannot be excluded. Residual confounding related to ICU interventions, including insulin therapy, nutritional support, and other aspects of neurocritical care management, also remains possible.

Second, some subgroup analyses were limited by sample size, particularly those involving HbA1c-derived measures. Because HbA1c measurements were available in only approximately one-quarter of the cohort, analyses involving the stress hyperglycemia ratio (SHR) should be considered exploratory and interpreted cautiously.

Third, functional outcome was only assessed at hospital discharge rather than at a standardized 90-day follow-up time point. As a result, discharge modified Rankin Scale scores may underestimate later neurological recovery, particularly among patients transferred to rehabilitation facilities.

Finally, although overall data completeness was high, a minority of earlier cases had reduced availability of longitudinal measurements because of historical data archiving limitations. Additionally, multiple correlated metabolic predictors were evaluated in separate models, which may increase the risk of type I error.

## 5. Conclusions

In patients with aSAH, longitudinal metabolic dysregulation was strongly associated with mortality and neurological outcomes. Persistent hyperglycemia, glycemic variability, and repeated GPR assessment provided prognostic information beyond isolated admission glucose measurements, with longitudinal GPR measures demonstrating particularly strong associations with mortality and functional disability.

The temporal pattern of associations suggests that early metabolic disturbances may be linked to DCI, whereas sustained abnormalities appear to reflect cumulative injury and increased risk of death or poor neurological outcome. These findings support the potential utility of longitudinal metabolic monitoring as a practical adjunct to established clinical assessment for dynamic risk stratification after aSAH. However, prospective multicenter studies are required to validate these observations and determine their therapeutic implications.

## Figures and Tables

**Table 1:** Baseline characteristics of the study cohort.

Characteristic	Overall *N* = 199	30-Day Survivors *N* = 164	30-Day Non-Survivors *N* = 35	*p*
Age, years	56.0 (47.0–66.0)	54.5 (45.0–65.5)	61.0 (53.0–68.0)	0.030
Sex				0.15
female	113 (56.8%)	97 (59.1%)	16 (45.7%)	
male	86 (43.2%)	67 (40.9%)	19 (54.3%)	
BMI	26.3 (23.4–29.7)	26.3 (23.4–30.2)	26.1 (23.4–29.4)	0.7
Hunt–Hess grade				<0.001
good (I–III)	112 (56.3%)	103 (62.8%)	9 (25.7%)	
poor (IV–V)	87 (43.7%)	61 (37.2%)	26 (74.3%)	
Glasgow Coma Scale	13 (7–14)	13 (7–15)	5 (4–13)	<0.001
Vasograde				<0.001
green	28 (14.1%)	27 (16.5%)	1 (2.9%)	
yellow	83 (41.7%)	76 (46.3%)	7 (20.0%)	
red	88 (44.2%)	61 (37.2%)	27 (77.1%)	
Admission values				
glucose, mmol/L	8.5 (7.0–10.4)	8.2 (6.6–9.9)	10.7 (9.1–13.2)	<0.001
GPR	2.3 (1.9–3.0)	2.2 (1.7–2.9)	2.9 (2.7–3.4)	<0.001
potassium, mmol/L	3.7 (3.3–4.1)	3.7 (3.3–4.1)	3.6 (3.3–4.1)	0.7
WBC, ×10^9^/L	13.8 (10.6–17.7)	13.7 (10.5–17.0)	14.9 (11.7–20.1)	0.083
CRP, mg/L	2.9 (1.3–8.4)	2.7 (1.3–8.9)	4.7 (1.6–7.3)	0.743
Diabetes mellitus	15 (7.5%)	13 (7.9%)	2 (5.7%)	1
Hypertension	92 (46.2%)	72 (43.9%)	20 (57.1%)	0.215
Current smoking	110 (55.3%)	90 (54.9%)	20 (57.1%)	0.8
Anterior aneurysm	168 (84.4%)	143 (87.2%)	25 (71.4%)	0.036
Posterior aneurysm	31 (15.6%)	21 (12.8%)	10 (28.6%)	0.036
Endovascular coiling	140 (70.4%)	114 (69.5%)	26 (74.3%)	0.685
Surgical clipping	59 (29.6%)	50 (30.5%)	9 (25.7%)	0.685

Continuous variables are reported as median values with interquartile ranges, whereas categorical variables are presented as counts and percentages. *p*-values correspond to comparisons between survivors and non-survivors. Abbreviations: BMI—body mass index; ICU—intensive care unit; WFNS—World Federation of Neurosurgical Societies; GCS—Glasgow Coma Scale; mRS—modified Rankin Score; glucose-potassium ratio—GPR; white blood cells—WBC; C-reactive protein—CRP.

**Table 2:** Association of glycemic and metabolic indices with 30-day mortality and poor functional outcome.

Variable	Mortality OR (95% CI)	*p*-value	Poor outcome OR (95% CI)	*p*-Value
GPR (admission)	1.53 (1.06–2.26)	0.025	1.95 (1.21–3.34)	0.010
GPR (EBI)	1.72 (1.16–2.64)	0.009	2.99 (1.77–5.36)	<0.001
GPR (30-day)	2.56 (1.66–4.16)	<0.001	2.90 (1.80–5.03)	<0.001
Glucose (admission)	1.63 (1.12–2.45)	0.012	2.25 (1.40–3.78)	0.001
Glucose (EBI)	1.84 (1.21–2.96)	0.008	3.22 (1.90–5.79)	<0.001
Glucose (30-day)	2.71 (1.74–4.45)	<0.001	3.50 (2.08–6.44)	<0.001
Glucose SD	1.58 (1.08–2.44)	0.026	1.93 (1.16–3.53)	0.022
Glucose range	1.15 (0.81–1.72)	0.429	2.22 (1.15–4.87)	0.035
Daily glucose change	1.80 (1.23–2.65)	0.002	1.65 (1.14–2.53)	0.012
Hyperglycemia days	1.10 (1.02–1.20)	0.020	1.39 (1.19–1.71)	<0.001

Odds ratios (OR) with 95% confidence intervals (CIs) were derived from multivariable logistic regression models adjusted for age, Glasgow Coma Scale (GCS), and Hunt–Hess grade. Continuous variables were standardized (per 1 SD increase), except hyperglycemia burden, which was analyzed per additional hyperglycemic day.

**Table 3:** Association of glucose–potassium ratio (GPR) with secondary clinical outcomes.

Outcome	Predictor	Effect	*p*-Value
DCI	GPR (EBI)	1.37 (1.00–1.93)	0.056
DND	GPR (30-day)	1.47 (1.08–2.07)	0.019
TCD vasospasm	GPR (30-day)	1.03 (0.77–1.39)	0.851
ICU neurological deficit	GPR (ICU)	2.06 (1.29–3.50)	0.004
ICU length of stay	GPR (ICU)	1.6% (-6.8% to 10.8%)	0.719
Hospital length of stay	GPR (30-day)	-6.5% (-15.8% to 4.0%)	0.214

Odds ratios and corresponding 95% confidence intervals were estimated using multivariable logistic regression models adjusted for age and Vasograde. Continuous variables were standardized per 1 SD increase. DCI = delayed cerebral ischemia; DND = delayed neurological deterioration; EBI = early brain injury (days 1–3); TCD = transcranial Doppler; ICU = intensive care unit.

## Data Availability

Anonymized data may be made available from the corresponding author upon reasonable request.

## Abbreviations

aSAH	aneurysmal subarachnoid hemorrhage
DCI	delayed cerebral ischemia
EBI	early brain injury
DND	delayed neurological deterioration
ICU	intensive care unit
GPR	glucose-potassium ratio
CT	computed tomography
CTA	computed tomography angiography
DSA	digital subtraction angiography
TCD	transcranial Doppler
BMI	body-mass index
GCS	Glasgow Coma Scale
WFNS	World Federation of Neurosurgical Societies
WBC	white blood cells
CRP	C-reactive protein
mRS	modified Rankin Scale
SD	standard deviation
CV	coefficient of variation
OR	odds ratio
CI	confidence interval
HR	hazard ratio
ROC	receiver operating characteristic
AUC	area under the curve
SHR	stress hyperglycemia ratio
